# Appraisal of long-time outcomes after curative surgery in elderly patients with gastric cancer: a propensity score matching analysis

**DOI:** 10.1186/s12893-021-01046-0

**Published:** 2021-01-09

**Authors:** Tomoyuki Matsunaga, Ryo Ishiguro, Wataru Miyauchi, Yuji Shishido, Kozo Miyatani, Manabu Yamamoto, Naruo Tokuyasu, Shuichi Takano, Teruhisa Sakamoto, Soichiro Honjo, Hiroaki Saito, Yoshiyuki Fujiwara

**Affiliations:** 1 Division of Gastrointestinal and Pediatric Surgery, Department of Surgery, School of Medicine, Tottori University Faculty of Medicine, Yonago, 683-8504 Japan; 2 Department of Surgery, Japanese Red Cross Tottori Hospital, Tottori, 680-8517 Japan

**Keywords:** Elderly patient, Gastric cancer, Prognosis

## Abstract

**Background:**

This study was conducted to assess the long-term outcomes of elderly patients among propensity-score-matched gastric cancer patients after curative gastrectomy and to propose the proper management of elderly gastric cancer patients.

**Methods:**

We enrolled 626 patients with gastric cancer who underwent curative gastrectomy at our institution between January 2004 and December 2015. To minimize selection bias among 2 groups, propensity score matching was performed.

**Results:**

Patients were divided into an elderly group over 75 years old (EP group; n = 186) and a non-elderly group (NEP group; n = 440). After propensity score matching, patients were divided into EP group (n = 178) and NEP group (n = 175). Five-year overall survival was significantly lower in the EP group than in the NEP group, consistent with a subgroup analysis of each stage. However, the 5-year disease-specific survival among all enrolled patients and those with stage I and II disease did not differ significantly. Moreover, in the subgroup of stage III patients, 5 year disease-specific survival was significantly lower in the EP group (23.0%) than in the NEP group (59.4%; *P* = 0.004). Because elderly patients with stage III disease had an extremely poor prognosis, we decided to compare the two groups with stage III. The EP group contained significantly fewer patients with D2 lymphadectomy (*P* = 0.002) and adjuvant chemotherapy (*P* < 0.001) than the NEP group. C-reactive protein to albumin ratio was significantly higher in patients in the EP group than in the NEP group (*P* = 0.046), and the prognostic nutritional index was significantly lower in patients in the EP group than in the NEP group (*P* = 0.045).　Multivariate analysis revealed that the prognostic nutritional index and lymphatic invasion were independent prognostic factors.

**Conclusions:**

Elderly gastric cancer patients with stage III disease showed poorer disease-specific survival compared with non-elderly patients, which may be due to a poorer nutritional and inflammatory background, fewer D2 lymphadenectomies, and a lack of adjuvant chemotherapy. The safe induction of standard lymphadenectomy and adjuvant chemotherapy with perioperative aggressive nutritional support may improve the prognosis of elderly gastric cancer patients with stage III disease.

## Background

Gastric cancer is the fourth most common cancer in the world and the second leading cause of cancer-related deaths, while the number of elderly gastric cancer patients is reported to be increasing [1, 2]. Advances in surgery, anesthesia, and pre- and post-operative management have led to an increase in gastrectomies conducted on elderly patients. Surgeons sometimes have difficulties in deciding upon the surgery of elderly patients because elderly patients are poorly nourished and have a variety of comorbidities [3]. For these reasons, although curative surgery with standard lymph node dissection is important in gastrectomy, surgeons often refrain from lymph node dissection because elderly patients might experience serious postoperative complications [4, 5]. However, there are few studies evaluated outcomes after operation of gastric cancer in elderly patients, and whether lymph node dissection is associated with poor cancer prognosis is unclear.

Adjuvant chemotherapy is necessary to improve survival in advanced gastric cancer patients after curative surgery [6]. The efficacy of adjuvant chemotherapy in gastric cancer has been demonstrated by various randomized control trials [6–8]. Chemotherapy is toxic and can cause serious side effects, while age is associated with increased toxicity and is considered a risk factor for reduced tolerance to chemotherapy. Despite the increase in elderly gastric cancer patient populations, there are few reports that indicate the efficacy of chemotherapy for elderly patients. Various clinical trials often enroll fewer elderly patients or exclude them altogether. The efficacy of limited lymph node dissection and adjuvant chemotherapy is unclear in patients with stage III disease where recurrence risk is high.

This study was conducted to assess the long-term outcomes of elderly patients among propensity-score-matched gastric cancer patients after curative gastrectomy and to propose the proper management of elderly gastric cancer patients.

## Methods

### Patients

This retrospective study was performed at Tottori University Hospital from January 2004 to December 2015, during which 626 gastric cancer patients underwent curative gastrectomy. The Japanese Gastric Cancer Treatment Guidelines were used to determine tumor status and the degree of lymph node dissection [9]. Patients with other primary cancers, distant metastases, and neoadjuvant chemotherapy were excluded from this study. Patients over 75 years old were defined as the elderly patient (EP) group, and patients under 75 years old were defined as the non-elderly patient (NEP) group. The modified frailty index (mFI) and Charlson comorbidity index (CCI) was calculated [10, 11]. To minimize selection bias among 2 groups, propensity score matching was performed with a logistic regression model and a 1:1 nearest neighbor-matching using MatchIt package on R version 3.6.3 software. The following variables were selected and matched as matching variables because these variables were determined to have a significant survival impact: sex (male, female), depth of tumor invasion (T1, T2, T3, T4), lymph node metastasis (N0, N1, N2, N3), lymphatic invasion (positive, negative), venous invasion (positive, negative), histologic type (differentiated, undifferentiated), type of gastrectomy (partial gastrectomy, total gastrectomy, proximal gastrectomy), and pathological stage (I, II, III). In addition, patients who had removed less than 16 lymph nodes were excluded from the analysis.

### Surgical procedures and postoperative management

Gastrectomy was performed with D2 lymph node dissection for advanced cancer and D1 + lymph node dissection for early gastric cancer according to the Japanese gastric cancer treatment guidelines [9]. However, the refrain of lymph node dissection for surgery in the elderly or high-risk patients was determined by physician. The indication for adjuvant chemotherapy is patients with pathological stage II and stage III disease excluding T3N0 [9]. The adjuvant chemotherapy was based on oral 5-fluorouracil derivatives without the combination of other agents. Indications for adjuvant chemotherapy, including elderly patients, were those with preserved organ function, Eastern Cooperative Oncology Group Performance Status 0 or 1, and adequate oral intake, and consent was obtained from each patient. Patients were periodically checked for recurrence via physical examination and blood tests every 3 months after discharge from the hospital. Computed tomography (CT) was performed at least every 6 months after surgery. The recurrence patterns and causes of death were examined from clinical records, CT, and positron emission tomography CT. In patients who were difficult to follow, we made direct enquires with their families.

### Definition of inflammation-based factors

The findings of peripheral blood tests, such as serum albumin level, total white blood cell count, total platelet count (PC), lymphocyte count (LC), and neutrophil count (NC) were collected from patients’ records. Preoperative blood tests were performed within 5 days before surgery. The platelet-to-lymphocyte ratio (PLR) and neutrophil-to-lymphocyte ratio (NLR) and were obtained by dividing the peripheral PC and NC by the peripheral LC, respectively [12]. The prognostic nutritional index (PNI) was calculated as follows: 10 × ALB concentration + 0.005 × total LC [13]. The CRP/ALB ratio (CAR) was calculated by dividing the CRP level by the ALB level (CRP measured in mg/L and albumin measured in g/L) [14]. The Youden index was calculated using receiver operating characteristic (ROC) analysis, to determine optimal cutoffs for CAR, NLR, and PNI in the 5-year disease-specific survival analysis.

### The definition of complications

The Clavien–Dindo (CD) system was used to determine postoperative complications [15]. In this study, postoperative complications were defined as those of CD classification grade II or more occurring within 30 days after surgery. If multiple complications occurred, a higher CD classification was used in the present study.

### Statistical analysis

Categorical variables were compared via χ^2^ test or Fisher’s exact tests. Mann–Whitney U test was used to compare continuous data, which was expressed as mean ± standard deviation. The time from the date of surgery until death from any cause, including death resulting from another disease, was defined as overall survival (OS). Survival curves were calculated using the Kaplan–Meier method, and differences between survival curves were examined using the log-rank test. Cox’s proportional hazards model was used for univariate and multivariate analyses of factors considered prognostic for disease-specific survival (DSS). *P* < 0.05 was considered significant. All reported statistical analyses were performed using JMP v9.0.1 software (SAS Institute, Inc., Cary, NC, USA).

## Results

### Patient characteristics

Overall, there were 450 (71.9%) male and 176 (28.1%) female patients, and their median age was 67.8 ± 11.5 years (range, 27–93). The pathological disease stages were I, II, and III in 432, 115, and 79 patients, respectively. Patients were divided into an elderly group over 75 years old (EP group; n = 186) and a non-elderly group (NEP group; n = 440). The relationships between the age and clinicopathological variables of the patients are shown in Table 1. As for histology, the EP group included more patients with differentiated-type carcinoma compared with the NEP group (*P* = 0.005). The EP group included significantly less patients with CCI low compared with the NEP group (*P* < 0.001), and mFI were significantly higher in patients in the EP group than in those in the NEP group (*P* < 0.001). Positive venous invasion was significantly higher in patients in the EP group than in those in the NEP group (*P* = 0.004). The EP group contained significantly fewer patients who underwent D2 lymphadectomy (*P* = 0.005) and adjuvant chemotherapy (*P* < 0.001) than the NEP group. Death from another disease was significantly higher in patients in the EP group than in those in the NEP group (*P* < 0.001). CAR and NLR were significantly higher in the EP group than in those in the NEP group (*P* < 0.001, *P* = 0.002, respectively). PNI was significantly lower in the EP group than in those in the NEP group (*P* < 0.001). No significant differences were observed regarding sex, tumor size, type of gastrectomy, approach, depth of tumor invasion, lymph node metastasis, lymphatic invasion, pathological stage, death from primary disease, and PLR. After propensity score matching, 19 patients (8 patients; EP group, 11 patients; NEP group) were excluded because lymph node had been dissected less than 16, and all excluded patients were stage I. Finally, 353 patients were selected for analysis. No significant differences were observed between the two groups except for age, CCI, mFI, lymphadectomy, adjuvant chemotherapy, death from another disease, CAR, NLR, and PNI (Table 1).

**Table 1:** Clinicopathological features of patients in the EP group and NEP group before and after propensity score matching

Characteristics	Before matching			After matching		
	EP group (n = 186)	NEP group (n = 440)	*p* value	EP group (n = 178)	NEP group (n = 175)	*p* value
Age (years)	80.0 ± 4.1	62.6 ± 9.5	< 0.001	80.2 ± 4.0	63.8 ± 9.0	< 0.001
Sex			0.206			0.473
Male	127 (68.3)	323 (73.4)		123 (69.1)	127 (72.6)	
Female	59 (31.7)	117 (26.6)		55 (30.9)	48 (27.4)	
CCI			< 0.001			0.002
Low	65 (34.9)	243 (55.2)		64 (36.0)	91 (52.0)	
Moderate/severe	121 (65.1)	197 (44.8)		114 (64.0)	84 (48.0)	
mFI	0.074 ± 0.068	0.046 ± 0.061	< 0.001	0.073 ± 0.067	0.050 ± 0.064	< 0.001
Number of analyzed lymph nodes			0.577			
< 16	8 (4.3)	21 (4.7)		0	0	
≥16	178 (95.7)	419 (95.3)		178	175	
Number of positive lymph nodes	1.75 ± 5.34	1.62 ± 5.90	0.546	1.70 ± 4.80	2.13 ± 8.04	0.738
Tumor size (mm)	40.4 ± 25.9	38.9 ± 25.6	0.470	40.5 ± 25.5	41.3 ± 26.3	
Depth of tumor invasion			0.479			0.956
T1	115 (61.8)	288 (65.5)		107 (60.1)	108 (61.7)	
T2	26 (14.0)	49 (11.1)		26 (14.6)	22 (12.6)	
T3	33 (17.7)	84 (19.1)		33 (18.5)	34 (19.4)	
T4	12 (6.5)	19 (4.3)		12 (6.8)	11 (6.3)	
Lymph node metastasis			0.765			0.700
Positive	51 (27.4)	112 (25.5)		49 (27.5)	46 (26.3)	
Negative	135 (72.6)	328 (74.5)		129 (72.5)	129 (73.7)	
Histologic type			0.005			0.511
Differentiated	116 (62.4)	220 (50.0)		111 (62.4)	115 (65.7)	
Undifferenciated	70 (37.6)	220 (50.0)		67 (37.6)	60 (34.3)	
Lymphatic invasion			0.136			0.845
Positive	110 (59.1)	231 (52.5)		106 (59.6)	106 (60.6)	
Negative	76 (40.9)	209 (47.5)		72 (40.4)	69 (39.4)	
Venous invasion			0.004			0.973
Positive	102 (54.8)	184 (41.8)		100 (56.2)	98 (56.0)	
Negative	84 (45.2)	256 (58.2)		78 (43.8)	77 (44.0)	
Stage of disease			0.550			0.578
I	124 (66.7)	308 (70.0)		116 (65.2)	119 (68.0)	
II	39 (21.0)	76 (17.2)		39 (21.9)	31 (17.7)	
III	23 (11.3)	56 (12.8)		23 (12.9)	25 (14.3)	
Type of gastrectomy			0.508			0.951
Distal	131 (70.4)	325 (73.9)		125 (70.2)	125 (71.4)	
Total	34 (18.3)	64 (14.5)		34 (19.1)	33 (18.9)	
Proximal	21 (11.3)	51 (11.6)		19 10.7)	17 (9.7)	
Approach			0.476			0.101
Open	71 (38.2)	183 (41.6)		67 (37.6)	82 (46.9)	
Laparo	115 (61.8)	257 (58.4)		111 (62.4)	93 (53.1)	
Lymphadectomy			0.005			0.006
< D2	136 (73.1)	293 (66.6)		130 (73.0)	103 (58.9)	
D2	50 (26.9)	147 (33.4)		48 (27.0)	72 (41.1)	
Adjuvant chemotherapy			< 0.001			< 0.001
Present	12 (6.5)	84 (19.1)		11 (6.2)	37 (21.1)	
Absent	174 (93.5)	356 (80.9)		167 (93.8)	138 (78.9)	
Death from another disease			< 0.001			0.022
Present	33 (17.7)	37 (8.4)		31 (17.4)	16 (9.1)	
Absent	153 (82.3)	403 (91.6)		147 (82.6)	159 (90.9)	
Death from primary disease			0.189			0.342
Present	23 (12.4)	39 (8.9)		23 (12.9)	17 (9.7)	
Absent	163 (87.6)	401 (91.1)		155 (87.1)	158 (90.3)	
CAR	0.127 ± 0.342	0.066 ± 0.176	< 0.001	0.121 ± 0.340	0.066 ± 0.164	0.005
NLR	2.797 ± 1.677	2.464 ± 1.597	0.002	2.778 ± 1.647	2.366 ± 1.159	0.013
PLR	167.1 ± 87.7	160.3 ± 85.3	0.327	168.2 ± 87.9	152.4 ± 66.5	0.114
PNI	46.4 ± 5.8	50.4 ± 5.6	< 0.001	46.5 ± 5.8	50.1 ± 5.1	< 0.001

Data are presented as the mean ± standard deviation or number (percentage) of patients

*CAR* C-reactive protein-to-albumin ratio, *CCI* Charlson comorbidity index, *EP* elderly patient, *mFI* modified frailty index, *NLR* neutrophil-to-lymphocyte ratio, *PLR* platelet-to-lymphocyte ratio, *PNI* prognostic nutritional index, *NEP* non-elderly patient

### Postoperative long-term outcomes

**Fig. 1:**
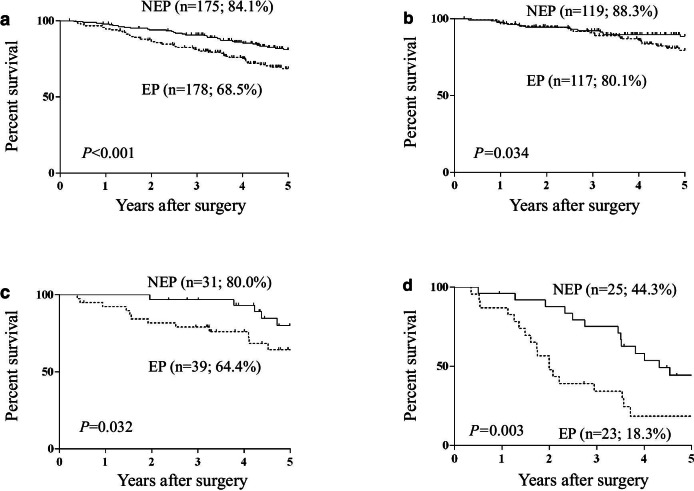
Overall survival curves of all patients (**a**), and those classified as stage I (**b**), stage II (**c**), and stage III (**d**). *EP* elderly patient, *NEP* non-elderly patient

**Fig. 2:**
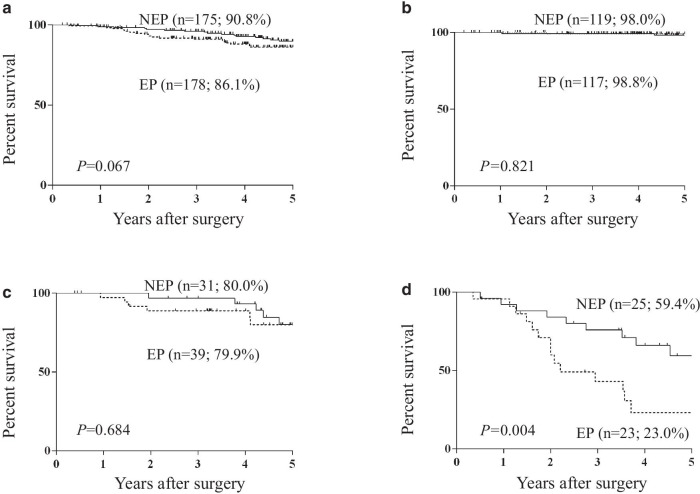
Disease-specific survival curves of all patients (**a**), and those classified as stage I (**b**), stage II (**c**), and stage III (**d**). *EP* elderly patient, *NEP* non-elderly patient

The 5-year OS rate was significantly lower in the EP group (68.5%) than in the NEP group (84.1%; *P* < 0.001; Fig. 1a) in all patients enrolled in this study. The significantly worse OS in the EP group was also observed in subgroups with stage I (Fig. 1b), stage II (Fig. 1c), and stage III (Fig. 1d) disease. However, the 5-year DSS among all enrolled patients and those with stage I and II disease did not differ significantly (all stage, *P* = 0.067, Fig. 2a; stage I, *P* = 0.821, Fig. 2b; stage II, *P* = 0.684, Fig. 2c). Moreover, in the subgroup of stage III patients, 5-year DSS was significantly lower in the EP group (23.0%) than in the NEP group (59.4%; *P* = 0.004, Fig. 2d).

### Patient characteristics in stage III disease

Because elderly patients with stage III disease had an extremely poor prognosis, we decided to compare the two groups with stage III. The clinicopathological characteristics of stage III patients are summarized in Table 2. No marked differences were observed in sex, CCI, mFI, tumor size, depth of tumor invasion, lymph node metastasis, histological type, lymphatic invasion, and venous invasion. The surgical procedure and short-term outcome in patients with stage III disease are shown in Table 3. The EP group contained significantly fewer patients who underwent D2 lymphadectomy (*P* = 0.015) and adjuvant chemotherapy (*P* < 0.001) than the NEP group. The number of analyzed lymph nodes were significantly lower in patients in the EP group than in those in the NEP group (*P* = 0.005). Death of primary disease was significantly higher in patients in the EP group than in those in the NEP group (*P* = 0.043). No marked differences were observed in the type of gastrectomy, approach, the number of positive lymph nodes, death from another disease or in the frequency of postoperative complications. In the EP group, 5-year DSS tended to be lower in patients who did not receive adjuvant chemotherapy (38.1%) than in those who received adjuvant therapy (11.3%), although the difference was not significant (P = 0.169).

**Table 2:** Clinicopathological characteristics of stage III patients

	EP group(n = 23)	NEP group(n = 25)	*p* value
Age (years)	80.5 ± 4.9	62.2 ± 8.92	< 0.001
Sex			0.075
Male	21 (91.3)	17 (68.0)	
Female	2 (8.7)	8 (32.0)	
CCI			0.067
Low	6 (26.1)	13 (52.0)	
Moderate/severe	17 (73.9)	12 (48.0)	
mFI	0.087 ± 0.068	0.051 ± 0.058	0.068
Tumor size (mm)	64.1 ± 27.5	69.9 ± 31.2	0.174
Depth of tumor invasion			0.548
T1	0	0	
T2	2 (8.7)	2 (8.0)	
T3	10 (43.5)	14 (56.0)	
T4	11 (47.8)	9 (36.0)	
Lymph node metastasis			0.845
N0	0	0	
N1	2 (8.7)	3 (12.0)	
N2	11 (47.8)	9 (36.0)	
N3	10 (43.5)	13 (52.0)	
Histologic type			1.000
Differentiated	8 (34.8)	8 (32.0)	
Undifferenciated	15 (65.2)	17 (68.0)	
Lymphatic invasion			0.214
ly0	0	0	
ly1	4 (17.4)	3 (12.0)	
ly2	8 (34.8)	15 (60.0)	
ly3	11 (47.8)	7 (28.0)	
Venous invasion			0.907
v0	2 (8.7)	3 (12.0)	
v1	9 (39.1)	10 (40.0)	
v2	9 (39.1)	10 (40.0)	
v3	3 (13.1)	2 (8.0)	

Data are presented as the mean ± standard deviation or number (percentage) of patients

*CCI* Charlson comorbidity index, *EP* elderly patient, *mFI* modified frailty index, *NEP* non-elderly patient

**Table 3:** Surgical procedures and short-term outcomes of stage III disease

	EP group (n = 23)	NEP group (n = 25)	p value
Type of gastrectomy			0.246
Distal	11 (47.8)	12 (48.0)	
Total	10 (43.5)	13 (52.0)	
Proximal	2 (8.7)	0	
Approach			
Laparoscopic	5 (21.7)	6 (24.0)	
Open	18 (78.3)	19 (76.0)	
Lymphadectomy			
< D2	13 (56.5)	3 (12.0)	
D2	10 (43.5)	22 (88.0)	
The numbers of analyzed lymph nodes	41.1 ± 22.7	55.8 ± 22.3	
The numbers of positive lymph nodes	9.74 ± 9.22	13.0 ± 17.6	
Adjuvant chemotherapy			
Present	7 (30.4)	20 (80.0)	
Absent	16 (69.6)	5 (20.0)	
Postoperative complication (CD≥2)			
Present	8 (34.8)	6 (24.0)	
Absent	15 (65.2)	19 (76.0)	
Postoperative complication (CD≥3)			
Present	5 (21.7)	5 (20.0)	
Absent	18 (78.3)	20 (80.0)	
Death from another disease			
Present	4 (17.4)	5 (20.0)	
Absent	19 (82.6)	20 (80.0)	
Death from primary disease			0.043
Present	15 (65.2)	9 (36.0)	
Absent	8 (34.8)	16 (64.0)	

Data are presented as number (percentage) of patients

*CD* Clavien–Dindo, *EP* elderly patient, *NEP* non-elderly patient

### Systemic inflammatory response in patients with Stage III

Clinical features including systemic inflammatory response in patients with stage III disease are shown in Table 4. CAR was significantly higher in patients in the EP group than in those in the NEP group (*P* = 0.046). Albumin and PNI were significantly lower in the EP group than in those in the NEP group (*P* = 0.036 and *P* = 0.045, respectively). No significant differences were observed regarding WBC, CRP, PC, NLR, and PLR.

**Table 4:** Systemic inflammatory response in patients with stage III disease

	EP group (n = 23)	NEP group (n = 25)	*P*-value
WBC	6796 ± 2069	6564 ± 2098	0.505
CRP	0.75 ± 1.51	0.43 ± 0.65	0.055
Albumin	3.73 ± 0.49	3.96 ± 0.44	0.036
PC	23.1 ± 7.2	26.3 ± 7.8	0.053
CAR	0.274 ± 0.683	0.115 ± 0.187	0.046
NLR	3.412 ± 2.074	2.902 ± 1.440	0.760
PLR	161.9 ± 64.9	181.9 ± 90.7	0.124
PNI	44.8 ± 5.5	48.0 ± 5.4	0.045

Data are presented as the mean ± standard deviation of patients

*CRP* C-reactive protein, *CAR* C-reactive protein-to-albumin ratio, *NLR* neutrophil-to-lymphocyte ratio, *PC* platelet count, *PLR* platelet-to-lymphocyte ratio, *PNI* prognostic nutritional index

### Univariate and multivariate analyses of patients with stage III disease

We performed univariate analysis of clinicopathological factors considered prognostic for DSS in patients with stage III disease. Univariate analysis identified age, lymphatic invasion, the number of positive lymph nodes, adjuvant chemotherapy, and PNI as prognostic indicators (Table 5). Then, in the multivariate analysis, we included parameters significant at *P* < 0.05 in the univariate analysis. Multivariate analysis revealed that PNI and lymphatic invasion were independent prognostic factors (Table 5).

**Table 5:** Univariate and multivariate analyses of prognostic factors for disease-specific survival in patients with stage III disease

	Univariate analysis			Mutivariate analysis		
	Hazard ratio	95% CI	*P* value	Hazard ratio	95% CI	*P* value
Age (≧75vs < 75)	3.200	1.377–7.437	0.007	1.754	0.670–4.594	0.252
Gender (Female vs Male)	0.728	0.268–1.978	0.534			
mFI (≧0.91 vs < 0.91)	1.296	0.573–2.930	0.534			
CCI (Moderate/severe vs Low)	1.017	0.453–2.282	0.967			
Lymphatic invasion (3 vs 0,1,2)	3.608	1.584–8.214	0.002	4.356	1.695–11.196	0.002
Venous invasion (2,3 vs 0,1)	1.241	0.555–2.773	0.599			
pT(4 vs 1,2,3)	2.229	0.992–5.010	0.052			
pN (2,3 vs 0,1)	1.683	0.741–3.820	0.213			
The number of positive lymph node (≥5 vs < 5)	3.517	1.045–11.832	0.042	2.429	0.688–8.567	0.168
Histologic type (Differenciated vs Undifferenciated	0.448	0.177–1.134	0.090			
Approach (Laparo vs Open)	1.513	0.625–3.662	0.359			
Type of gastrectomy (TG vs DG/PG)	1.525	0.674–3.450	0.311			
Lymphadectomy (≥D2 vs < D2)	0.501	0.221–1.136	0.098			
Adjuvant chemotherapy (Present vs Absent)	0.469	0.211–0.963	0.049	0.682	0.271–1.512	0.402
Postoperative complication (CD3 ≥vs < 3)	1.984	0.589–6.689	0.269			
CAR (≥0.064 vs < 0.064)	0.577	0.229–1.458	0.245			
NLR (≥2.273 vs < 2.273)	1.186	0.526–2.675	0.681			
PNI (≥49.8 vs < 49.8)	2.760	1.217–8.522	0.046	3.321	1.116–10.856	0.047

*CD* Clavien–Dindo, *CI* confidence interval, *DG* distal gastrectomy, *EP* elderly patient, *NEP* non-elderly patient, *PG* proximal gastrectomy, *pT* pathological depth of invasion, *pN* pathological lymph node metastasis, *TG* total gastrectomy

### Site of recurrence in patients with stage III disease

The site of recurrence was reviewed in patients with stage III disease, and hematogenous metastasis was significantly more frequent in the EP group than in the NEP group (*P* = 0.020; Table 6). No significant differences were observed regarding peritoneal metastasis, lymph node metastasis, and local recurrence (*P* = 0.703, *P* = 1.000, and *P* = 0.479, respectively; Table 6). Table 7 indicate the risk factor of hematogenous metastasis. Hematogenous metastasis tend to be less frequent in patients who underwent D2 lymphadectomy, although the difference was not significant (P = 0.055).

**Table 6:** Site of recurrence in patients with stage III disease

	EP group (n = 23)	NEP group (n = 25)	*P* value
Peritoneal metastasis			0.703
Present	3 (13.0)	5 (20.0)	
Absent	20 (87.0)	20 (80.0)	
Hematogenous metastasis			0.020
Present	7 (30.4)	1 (4.0)	
Absent	16 (69.6)	24 (96.0)	
Lymph node metastasis			1.000
Present	5 (21.7)	5 (20.0)	
Absent	18 (78.3)	20 (80.0)	
Local recurrence			0.479
Present	1 (4.3)	0 (0)	
Absent	22 (95.7)	25 (100)	

Data are presented as number (percentage) of patients

*EP* elderly patient, *NEP* non-elderly patient

**Table 7:** Risk factor of hematogenous metastasis in patients with stage III disease

	Hematogenous metastasis		*p* value
	Present (n = 8)	Absent (n = 40)	
Depth of tumor invasion			0.600
T2/3	4 (50.0)	24 (60.0)	
T4	4 (50.0)	16 (40.0)	
Lymph node metastasis			0.897
N1/2	4 (50.0)	21 (52.5)	
N3	4 (50.0)	19 (47.5)	
Histologic type			0.273
Differentiated	4 (50.0)	12 (30.0)	
Undifferenciated	4 (50.0)	28 (70.0)	
Lymphatic invasion			1.000
ly0/1/2	5 (62.5)	25 (62.5)	
ly3	3 (37.5)	15 (37.5)	
Venous invasion			0.439
v0/1	3 (37.5)	21 (52.5)	
v2/3	5 (62.5)	19 (47.5)	
Lymphadectomy			0.055
< D2	5 (62.5)	11 (27.5)	
D2	3 (37.5)	29 (72.5)	
Adjuvant chemotherapy			0.242
Present	3 (37.5)	24 (60.0)	
Absent	5 (62.5)	16 (40.0)	

Data are presented as number (percentage) of patients

## Discussion

In the present study, the 5-year OS rate was significantly lower in the EP group than in that in the NEP group. The 5-year DSS rates were significantly lower in stage III patients in the EP group than in those in the NEP group. CAR was significantly higher in patients in the EP group than in the NEP group, and PNI was significantly lower in patients in the EP group than in the NEP group. The EP group contained significantly fewer patients with D2 lymphadectomy and adjuvant chemotherapy than the NEP group. Multivariate analysis revealed that PNI and lymphatic invasion were independent prognostic factors in patients with stage III disease.

In this study, the 5-year OS rate was significantly poor in the EP group. This result was similar with recent reports that elderly patients have poor physical or nutritional statuses and some degree of frailty, and are likely to die from other diseases even if gastric cancer can be cured by gastrectomy [16, 17]. In this study, the 5-year DSS rate was significantly poor only in stage III EP group patients. However, the reason for the poor prognosis in elderly stage III gastric cancer patients has not been sufficiently elucidated, but one possible explanation is the low rate of PNI in stage III EP group patients. Sakurai et al. reviewed the prognosis of 147 elderly gastric cancer patients who underwent curative gastrectomy and showed that low preoperative PNI predicts the poor survival of patients with gastric cancer [18]. Park et al. also reviewed the prognosis of 1868 gastric cancer patients with Stage II/III who underwent gastrectomy and showed that low preoperative PNI predicts the poor survival. However, the reason for the independently significant correlation between preoperative PNI and postoperative DSS in patients with stage III is unknown. Previous studies have suggested that the inflammatory response in cancer patients is closely related to serum albumin levels and lymphocyte counts [19], and also suggest the systemic inflammatory response plays an important role in cancer development and progression [20]. Lymphocytes play an important role in immunity against tumors, and a decrease in lymphocyte count reflects a decrease in cellular immunity against cancer cells [20, 21]. In addition, proinflammatory cytokines have been reported to decrease serum albumin production in hepatocytes and reduce serum albumin levels [22]. Therefore, PNI may be associated with prognosis because it precisely reflects systemic inflammation, and a low PNI may indicate the possibility of a high-grade malignancy. Several studies have reported that preoperative CAR and NLR elevation have been reported to be long-term poor prognostic factors for gastric cancer [14, 23, 24], but these were not found to be independent prognostic factors in stage III EP group patients in the present study.

Another possible explanation of the poor prognosis is the low rate of adjuvant chemotherapy in this population. Surgery is the mainstay in patients with gastric cancer, but the prognosis is still poor in patients with far-advanced gastric cancer such as stage III disease [6]. Therefore, adjuvant therapy may contribute to the improved survival of patients with curative gastrectomy [25]. However, side effects can result from chemotherapy and can sometimes be severe. Furthermore, age is considered a risk factor for increased toxicity and poorer tolerance to chemotherapy. Ying et al. reported the survival benefits of adjuvant fluoropyrimidine-based chemotherapy among elderly patients with non-metastatic gastric cancer after D2 gastrectomy [26]. They reviewed the prognosis of 360 gastric cancer patients aged 65 years or older with non-metastatic gastric cancer who had undergone D2 gastrectomy, and showed that significant survival benefits were achieved with adjuvant chemotherapy in stage III patients but not in stage I or stage II patients. Mustafa et al. also reported that the addition of adjuvant chemotherapy after gastrectomy influenced survival in gastric cancer patients of ≥ 65 years of age [27]. Based on these reports, aggressive adjuvant chemotherapy may be required even in elderly gastric cancer patients with stage III disease.

In this study, patients in the EP group underwent significantly fewer D2 lymphadenectomies than the NEP group and the analyzed lymph node in patients in the EP group was significantly lower than those in the NEP group. The limited lymph node dissection in the elderly patients is another potential explanation of the poor prognosis. Complete tumor resection is essential to treat gastric cancer, and D2 gastrectomy is the standard surgical procedure for patients with advanced gastric cancer in Japan [9]. Ilfelt et al. reported that the limiting the extent of lymph node dissection affect not only lymph node recurrence but also another recurrence pattern [28]. They reviewed 711 patients who underwent curative gastrectomy, and assessed the effect of D2 compared with D1 surgery on disease recurrence and survival. They showed that regional recurrence and liver metastases were more common in the D1 group. In this study, the hematogenous metastasis was significantly more frequent in the EP group than in the NEP group, and hematogenous metastasis tend to be less frequent in patients who underwent D2 lymphadectomy, although the difference was not significant. Therefore, aggressive D2 lymph node dissection might lead to the better prognosis.

This study had several limitations. First, this was a retrospective study that used patients’ records from a single institution. Propensity score matching was used to balance the two groups, but the results must be interpreted carefully. Second, there are various definitions of the elderly patient [16, 29]. Currently, the Japanese Geriatrics Society has proposed to redefine the elderly as 75 years of age or older, and thus we adopted a threshold of 75 years [30]. Further well-designed multicenter prospective studies with larger populations are needed to confirm these findings.

## Conclusion

In conclusion, elderly gastric cancer patients with stage III disease showed poorer DSS compared with non-elderly patients, which may be due to a poorer nutritional and inflammatory background, fewer D2 lymphadenectomies, and a lack of adjuvant chemotherapy. The safe induction of standard lymphadenectomy and adjuvant chemotherapy with perioperative aggressive nutritional support may improve the prognosis of elderly gastric cancer patients.

## Data Availability

The datasets used and analyzed during the current study are available from the corresponding author on reasonable request.

## Abbreviations

CAR: C-reactive protein-to-albumin ratio
CCI: Charlson comorbidity index
CD: Clavien–Dindo
CRP: C-reactive protein
CT: Computed tomography
DSS: Disease-specific survival
EP: Elderly patient
mFI: Modified frailty index
NEP: Non-elderly patient
NLR: Neutrophil-to-lymphocyte ratio
PC: Platelet count
PLR: Platelet-to-lymphocyte ratio
OS: Overall survival
PNI: Prognostic nutritional index
